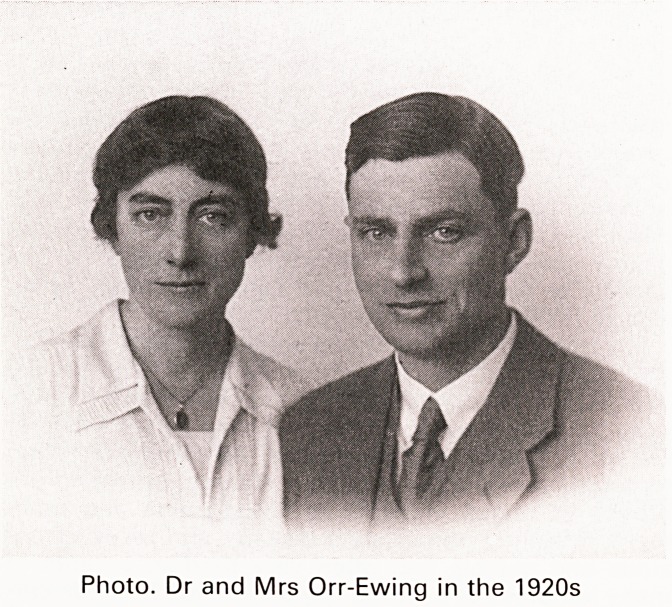# Dr H. J. Orr-Ewing

**Published:** 1987-08

**Authors:** C. B. Perry


### Obituary

### Dr Hugh James Orr-Ewing, MD, FRCP.

Photo. Dr and Mrs Orr-Ewing in the 1920s

Dr Hugh Orr-Ewing who died on March 8th a few weeks before his 96th birthday must have been one of the few surviving students of University College, Bristol. On the occasion of the celebration of the Centenary of the College in 1976 he represented the alumni at the official ceremony. Entering the Bristol Medical School in 1908 he studied for the London MB, BS externally, he graduated in 1913 with distinction in all the clinical subjects and was awarded the Gold Medal. In the same year he passed the Conjoint examination. He held house appointments at the Royal Infirmary and in one of his reminiscences he described the inadequate accommodation for residents and how common it was to see two of them in the same bath at the same time. At the outbreak of the 1914–18 war he enlisted in the RAMC and served in France until 1916 when he was invalided home having been awarded the military cross at the battle of Loos. In 1917 Orr Ewing passed the London MD examination and was again awarded the gold medal. The following year he became MRCP.

A devout Christian and an active lay reader for nearly 60 years he became a medical missionary in 1920 and was appointed medical superintendant of the English Mission Hospital in Jerusalem. Here he served for nearly twelve years and was appointed Honorary Consultant Physician to the Government of Palestine. An accomplished linguist it was reported that in dealing with patients in Jerusalem he might, in any one day, talk in Arabic, French, German, Spanish, Yiddish and Hebrew. In these posts he gained great distinction and was for many years remembered in Jerusalem with admiration and affection. He was elected FRCP in 1931.

Returning to England in 1934 he was appointed Assistant Physician to the Bristol General Hospital and at the same time became physician to Clifton College. He became full physician in 1945. Orr-Ewing was President of the old Bath, Bristol and Somerset Branch of the BMA 1946–1947 and gave his presidential address on "The history of Arabian Medicine". Three years later he was President of the Bristol Medico-Chirurgical Society. His presidential address on "Medicine in 18th Century Bristol" was published in the Journal (1950, vol 67).

Retiring from the Hospital in 1956 Orr-Ewing continued to lead an active life. He served on numerous committees, many connected with the church and claimed to have been a member of 50 and chairman of 13. He was a keen golfer but sadly this activity was somewhat hampered some years ago by an accident which resulted in the loss of one eye. This he bore with great bravery and fortitude as he did also his gradually increasing deafness. He was a gifted raconteur with a never ending fund of stories, one of his surgical colleagues once alleged that all the best of these started "When I was in Palestine. . . .". Before his death he made a tape recording which he wished played at his funeral in which he affirmed his Christian faith and his confident belief in a future life.

C. B. Perry

## Figures and Tables

**Figure f1:**